# Actin Interacts with Dengue Virus 2 and 4 Envelope Proteins

**DOI:** 10.1371/journal.pone.0151951

**Published:** 2016-03-24

**Authors:** Kunlakanya Jitoboam, Narumon Phaonakrop, Sirikwan Libsittikul, Chutima Thepparit, Sittiruk Roytrakul, Duncan R. Smith

**Affiliations:** 1 Institute of Molecular Biosciences, Mahidol University, Salaya campus, 25/25 Phuttamonton Sai 4, Salaya, Nakorn Pathom, Thailand; 2 National Center for Genetic Engineering and Biotechnology (BIOTEC), National Science and Technology Development Agency, 113 Thailand Science Park, Phahonyothin Road, Khlong Nueng, Khlong Luang, Pathum Thani, Thailand; 3 Center for Emerging and Neglected Infectious Diseases, Mahidol University, Salaya campus, 25/25 Phuttamonton Sai 4, Salaya, Nakorn Pathom, Thailand; Utah State University, UNITED STATES

## Abstract

Dengue virus (DENV) remains a significant public health problem in many tropical and sub-tropical countries worldwide. The DENV envelope (E) protein is the major antigenic determinant and the protein that mediates receptor binding and endosomal fusion. In contrast to some other DENV proteins, relatively few cellular interacting proteins have been identified. To address this issue a co-immuoprecipitation strategy was employed. The predominant co-immunoprecipitating proteins identified were actin and actin related proteins, however the results suggested that actin was the only *bona fide* interacting partner. Actin was shown to interact with the E protein of DENV 2 and 4, and the interaction between actin and DENV E protein was shown to occur in a truncated DENV consisting of only domains I and II. Actin was shown to decrease during infection, but this was not associated with a decrease in gene transcription. Actin-related proteins also showed a decrease in expression during infection that was not transcriptionally regulated. Cytoskeletal reorganization was not observed during infection, suggesting that the interaction between actin and E protein has a cell type specific component.

## Introduction

Dengue virus (DENV) is the most common cause of arthropod-borne viral infection in tropical and subtropical countries [1]. It is estimated that 390 million people get infected annually and approximately 96 million develop symptoms resulting from the infection [2]. DENV is an enveloped positive sense single stranded RNA virus belonging to the family *Flaviviridae*, genus *Flavivirus*. There are four antigenically distinct DENVs each of which can cause disease in humans ranging from a self-limited febrile illness known as dengue fever (DF) to more severe hemorrhagic fever (DHF) and dengue shock syndrome (DSS) [3]. The DENV virion is composed of three structural proteins (capsid (C), membrane (M) and envelope protein (E)) and an approximately 11 kb RNA genome which encodes the three structural proteins (C, M, and E protein) and seven non-structural proteins (NS1, NS2A, NS2B, NS3, NS4A, NS4B, and NS5) [4].

The DENV E protein is a glycoprotein that is comprised of three domains: domain I, the central N-terminal domain; domain II, the homodimerization domain responsible for endosomal membrane fusion; and domain III, the immunoglobulin-like domain responsible for target cell binding and viral entry [5, 6]. In order to infect host cells, the DENV E protein interacts with host cell receptors, followed by receptor-mediated endocytosis [7, 8]. Several studies have identified and characterized cell surface receptors in both insect and mammalian cells such as 36/67 kDa laminin high-affinity receptor [9], GRP78 [10, 11], heat shock protein 70 [12] and prohibitin [13].

Although the primary characterized function of the E protein is receptor binding during viral entry and subsequent membrane fusion, the limited genetic capacity of DENV suggests that the viral E protein might have multiple functions, including interacting with host cellular proteins to facilitate the viral replication cycle, especially in host immune defense alteration and manipulation of cellular pathways to create a more favorable environment for viral replication [14]. In this regard, several cellular proteins have been identified as DENV E protein intracellular interacting proteins, including the ER-resident chaperones BiP (GRP78), calnexin and calreticulin, which are important for infectious DENV virion production [15], with the latter two proteins having been described as having roles in viral glycoprotein processing and maturation [16, 17]. Thus identification of the interactions between DENV E and host cellular proteins will provide a better understanding of the mechanism of viral replication and pathogenesis, as well as identifying cellular pathways that might be used as targets for anti-dengue therapy development.

## Materials and Methods

### Cells and viruses

The human embryonic kidney cell line HEK239T/17 (ATCC CRL-11268) was cultivated in Dulbecco’s modified eagle’s medium (DMEM; Gibco Invitrogen, Carlsbad, CA) supplemented with 10% heat-inactivated fetal bovine serum (FBS, Gibco Invitrogen) without antibiotics at 37°C in a humidified incubator with 5% CO_2_. The *Aedes albopitus* cell line C6/36 (ATCC CRL-1660) was cultivated in minimum essential medium (MEM; Gibco Invitrogen) supplemented with 10% heat inactivated FBS, 100 units/ml of penicillin and 100 μg/ml of streptomycin (PAA Laboratories, Linz, Austria) at 28°C. The rhesus monkey kidney cell line LLC-MK_2_ (ATCC CCL-7) was cultivated in DMEM (Gibco Invitrogen) supplemented with 5% heat inactivated FBS and 100 units/ml of penicillin and 100 μg/ml of streptomycin at 37°C in a humidified incubator with 5% CO_2_. Dengue virus serotype 2 (strain 16681) and dengue virus serotype 4 (strain 1036), were propagated in C6/36 cells. Supernatants containing viruses were harvested by centrifugation to remove cell debris and store at -80°C. The viral titer was determined by standard plaque assay using LLC-MK_2_ cells as described elsewhere [18].

### Virus infection

On the day prior infection, HEK293T/17 cells were seeded into culture plates under standard growth conditions which allowed 70–80% confluence to be reached within 24 hrs. After 24 hrs of cultivation, culture medium was removed and the cells were incubated with DENV 2 or DENV 4 in DMEM medium at the indicated multiplicity of infection (m.o.i.) or with only DMEM medium for mock-infection for 2 hours. Then the medium containing the virus was removed and the cells were further incubated under standard condition for the times indicated.

### Flow cytometry

DENV infected cells was determined by flow cytometry as described elsewhere [19]. Briefly DENV infected or mock infected cells were incubated with a pan specific anti-dengue E protein and appropriate dilution of a secondary antibody conjugated with FITC. Then the cells were analyzed on a BD FACalibur cytometer (Becton Dickinson, BD Biosciences, San Jose, CA). Data analysis was performed using the CellQuest^™^ software.

### Cell viability analysis

HEK293T/17 cells were seeded into 6-well culture plates under standard condition which allowed 80% confluence to be reached within 24 hrs. After that the cells were infected with DENV 2 or DENV 4 in DMEM medium at the indicated m.o.i. or with DMEM medium for mock infection. Subsequently, the medium containing virus was removed and the cells were further grown under standard condition for the times indicated and cell viability determined by staining with 0.4% trypan blue and subsequent counting of viable cells under an inverted microscope. Experiment was undertaken independently in triplicate.

### Immunoprecipitation and protein identification

For immunoprecipitation experiments undertaken with viral infection, HEK293T/17 cells were seeded into 100 mm^2^ tissue culture plates at a density that allowed 70% confluence to be reached within 24 hrs after which cells were mock-infected or infected with DENV 2 at m.o.i. 5 or DENV 4 at m.o.i. 20 for 2 hrs. After removal of the viral inoculums, the cells were further cultured with complete DMEM medium for 2 days post infection.

For immunoprecipitation experiments using transfected constructs, HEK293T/17 cells were grown to 40–50% confluence in 100 mm^2^ tissue culture plates and mock-transfected or transfected with pcDNA-FLAG_D2ET, pcDNA-FLAG_D4ET or pcDNA-EGFP plasmids using the calcium phosphate mediated transfection method. The cells were further cultured for 2 days post-transfection.

Plates of infected cells or transfected cells were washed once with phosphate-buffer saline (PBS) and the cells were resuspended in pre-cooled lysis buffer (20 mM Tris–HCl pH 7.5, 150 mM NaCl, 1 mM EDTA, 1% Triton X-100, 2.5 mM sodium pyrophosphate, 1 mM β-glycerophosphate, 1 mM Na3VO4, 1 mM PMSF). Subsequently, the cells were lysed by sonicating at 4°C followed by centrifugation at 16000g for 5 min to remove cell debris. The supernatant was transferred into new tube and protein concentration was determined using the Bradford assay. Pre-clearing of lysates was performed by incubation of 1 mg of lysates with Protein G Sepharose 4 Fast Flow media (GE Healthcare, Buckinghamshire, UK) at 4°C and with rotatation for 1 hr. Subsequently, 100 μl of pre-cleared lysates from infections or mock-infections were incubated with or without 1 μg of a mouse pan specific anti-dengue E protein antibody (MAB8705, Millipore, MA) or 1 μg of a goat anti-actin antibody (sc-1616, Santa Cruz Biotechnology Inc.), while pre-cleared lysates from transfections or mock transfections were incubated with or without 1 μg of a goat anti-actin antibody (sc-1616, Santa Cruz Biotechnology Inc.) with gentle rocking overnight at 4°C. Subsequently 20 μl of protein G slurry was added to the solutions which were further incubated with gentle rocking at 4°C for 4 hrs. The samples were centrifuges at 6000 ×g for 5 min and the supernatants were discarded. The pellets were washed for four times with IP-lysis buffer and resuspended in 30 μl of 3×SDS sample loading buffer and heated to 100°C for 5 min followed by centrifugation at 14000×g for 3 min in order to elute protein complexes from the beads. The samples were applied to 12% or 15% SDS-polyacrylamide gels for electrophoresis. Gels were subsequently stained by silver staining (reference) and scanned before storage in deionized water or 0.1% acetic acid in deionized water at 4°C. Bands of interest were subsequently subjected to LC-MS and proteins identified by search of the NCBI nr database using the Mascot database search engine (Matrix Science, London, UK, (Perkins et al., 1999) exactly as described previously [20].

### Western blot analysis

Proteins were separated by electrophoresis through 12% SDS polyacrylamide gels followed by transfer to nitrocellulose membranes (GE Healthcare, Buckinghamshire, UK). The membranes were subsequently blocked with 5% skim milk in TBS/0.1% Tween 20 for 2 hours at room temperature. The membranes were incubated with a 1:500 dilution of mouse monoclonal anti-dengue serotype1-4 antibody (MA1-27093, Thermo Scientific, MA), a 1:500 dilution of goat anti-actin antibody (sc-1616, Santa Cruz Biotechnology Inc.), a 1:500 dilution of mouse monoclonal anti-dengue serotype1-4 antibody (MA1-27093, Thermo Scientific, MA), a 1:2000 dilution of rabbit polyclonal anti-OctA-Probe (Flag) antibody (sc-807; Santa Cruz Biotechnology Inc.), a 1:500 dilution of goat polyclonal anti-Hsp 27 antibody (sc-1048; Santa Cruz Biotechnology Inc.) or a 1:1000 dilution of rabbit polyclonal anti-myosin Ic antibody (sc-130177; Santa Cruz Biotechnology Inc.) at 4°C with shaking for overnight. For glyceraldehyde 3-phosphate dehydrogenase (GAPDH), the membranes were incubated with a 1:5000 dilution of mouse anti-GAPDH monoclonal antibody (sc-32233; Santa Cruz Biotechnology Inc.) for 1 hr at room temperature. The membranes were further incubated for 2 hours at room temperature with a 1:5000 dilution of a horseradish peroxidase (HRP) conjugated goat anti-mouse IgG, a 1:5000 dilution of a HRP-conjugated rabbit anti-goat IgG or a 1:8000 dilution of a HRP-conjugated goat anti-rabbit IgG as appropriate. The antigen-antibody complexes were detected using the Amersham ECL Prime Western Blotting Detection Reagent (GE Healthcare, Buckinghamshire, UK) and exposed membranes were exposed to autoradiography films for an appropriate period of time or were directly observed using ae visible western blot imaging system (Azure c400, Azure Biosystems, Inc., Dublin, CA).

### DENV E protein eukaryotic expression constructs

Total RNA form DENV 2 and DENV 4 stock viruses were extracted using TRI reagent (Molecular Research Center, Inc., Cincinnati, OH) according to the manufacturer’s instructions. First strand cDNA was synthesized using ImProm-II^™^ reverse transcriptase (Promega, Madison, WI) and random hexamers. cDNAs were amplified with specific primers to generate DENV 2 or DENV 4 E fragments corresponding to domain I, II and III of DENV E protein but lacking the transmembrane domain (D2ET, D4ET) or to generate a construct containing only domains I and II (D2E12). Primers used were (D2ET forward) 5’-ATGCGTTGCATAGGAATGTC -3’, (D2ET reverse) 5’-GCTCAACTGGTTTAAGAAATAACTCGAGCGC -3’, (D4ET forward) 5’–ATGCGATGCGTAGGAGTAGG -3’ and (D4ET reverse) 5’- CTCCATTGGTTCAGGAAATAACTCGAG -3’ and (D2E12 forward) 5’-CGGCTAGCCACCATGgattacaaggatgacgacgataagATGCG -3’ (lower case letters represent the FLAG peptide: DYKDDDDK) and (D2E12 reverse) 5’-CCGAATTCTTTGAGCTGTAGCTTGTTC -3’. The cDNA fragments (D2ET and D4ET) were ligated into the pRSET-B expression vector which provided an N-terminal six-histidine tag, generating pRSETB-D2ET and pRSETB-D4ET or (D2E12) directly into the pcDNA 3.1+ eukaryotic expression plasmid (Invitrogen, CA).

**Fig 1:**
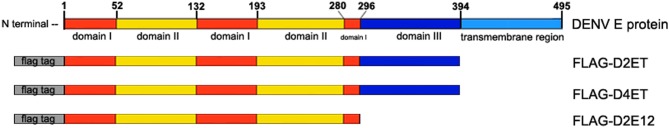
Diagrammatic representation of dengue virus E protein showing the three domains of this protein (domains I, II and III) as well as representation of the three constructs generated in this study.

pRSETB-D2ET and pRSETB-D4ET were subsequently used as templates to generate Flag-tagged constructs using primers FLAG-D2ET(forward) 5’-CGGCTAGCCACCATGgattacaaggatgacgacgataagATGCGTTGCATAGGAATGTC-3’; FLAG-D2ET(reverse) 5’-CGTGAATTCCTATTTCTTAAACCAGTTGAGCTTCAGTTGTCC-3’; FLAG-D4ET(forward) 5’-CGGCTAGCCACCATGgattacaaggatga cgacgataagATGCGATGCGTAGGAGTAGG-3’ and FLG-D4ET(reverse) 5’- GCTAAGCTTCTATTTCCTGAACCAATGGAGTG-3. Lower case letters denote the FLAG tag (DYKDDDDK) peptide. Cycle conditions were 98°C for 30s, and 30 cycles of 98°C for 30s, 30s of 63°C for flag peptide-tagged D2ET (FLAG-D2ET) or 60°C for flag peptide-tagged D4ET (FLAG-D4ET) and 72°C for 40s and final extension at 72°C for 5 min with Phusion DNA polymerase (Thermo Scientific, MA). The constructs were subsequently cloned into the pcDNA 3.1+ eukaryotic expression plasmid (Invitrogen, CA), generating the recombinant plasmids pcDNA-FLAG_D2ET and pcDNA-FLAG_D4ET. All constructs were verified by commercial DNA sequencing (Macrogen Inc., Korea). Constructs are shown in Fig 1.

### Immunofluorescence assay

HEK293T/17 cells were seeded on glass coverslips at a density that allowed 40–50% confluence to be reached within 24 hours following which the cells were infected with DENV 2 at m.o.i. 5 or DENV 4 at m.o.i. 20 for 2 hrs or mock infected and further cultured under standard conditions until required. For transfection experiments cells were transfected with pcDNA-FLAG_D2ET or pcDNA-FLAG_D4ET plasmids by calcium phosphate mediated transfection. Infected cells were collect at 0, 20, 30 min, 1 hr, 12, 24, 48 and 72 hrs post infection as indicated, while transfected cells were collected at 48 hrs post transfection. The cells were washed with PBS and fixed with 4% paraformaldehyde for 20 min. Consequently, the cells were permeabilized with 0.3% Triton X-100 in PBS for 10 min and blocked with 5% BSA in PBS for 10 min. Subsequently infected cells were incubated with a 1:100 dilution of a mouse monoclonal pan specific anti-dengue E protein antibody (MAB8705, Millipore, MA) while transfected cells were incubated with a 1:100 dilution of rabbit polyclonal anti-OctA-Probe (Flag) antibody (sc-807; Santa Cruz Biotechnology Inc.) at 4°C overnight. After primary antibody incubation, infected cells were washed with 0.03% Triton-X 100 in PBS four times followed by incubation at room temperature of infected cells with a 1:200 dilution of an Alexa 488-conjugated donkey anti-mouse IgG polyclonal antibody (A21202, Molecular Probes, Thermo Fisher Scientific), a 1:75 dilution of phalloidin-tetramethyl rhodamine B isothiocyanate (TRITC) for actin detection (Sigma-Aldrich, St. Louis, MO) and a 1:500 dilution of 4’,6-diamidino-2-phynyllindole (DAPI) (Calbiochem, EMD Chemical, Inc.). Transfected cells were incubated with a 1:100 dilution of an Alexa 647-conjugated donkey anti-rabbit IgG polyclonal antibody (A31573, Molecular Probes, Thermo Fisher Scientific) and DAPI. After washing all cells were mounted onto glass slides using Prolong^®^ Gold antifade reagent. Slides were subsequently observed under an Olympus Fluo View 1000 confocal microscope (Olympus, Shinjuku-ku, Tokyo, Japan) equipped with Olympus Fluo View Software version 4.0. For co-localization analysis, the Pearson correlation coefficients were determined using the ImageJ analysis program [21] and the JACoP plugin [22] as described elsewhere [23]. Results are represented in the term of Pearson correlation coefficients, with standard deviation (SD) and confidence levels (CIs).

### Semi-quantitative RT-PCR

DENV 2 or DENV 4 infected or mock infected cells were collected at 3, 6, 12, 24 and 72 hrs post infection. Total RNA was extracted using TRI reagent (Molecular Research Center, Inc., Cincinnati, OH) according to the manufacturer’s instructions and RNA concentrations were determined by spectrophotometry. The first strand cDNA was synthesized using ImProm-II^™^ reverse transcriptase (Promega, Madison, WI) and oligo(dT) primer. Then cDNA amplifications for actin, HSP27, MYOIC (myosin Ic) and GAPDH were performed with specific primers; β-Actin-F: 5’-GAAGATGACCCAGAT CATGT-3’, β -Actin-R: 5’- ATCTCTTGCTCGAAGTCCAG-3’; HSP27-F: 5’- TACATCTCCCGGTGCTTCA-3’), HSP27-R: 5’- AGGTGACTGGGATGGTGAT-3’; MYOIC-F: 5’- GGGAACCCGTCCAGTATTTC-3’, MYOIC-R: 5’- CTTGACAGTATCCTCCAGCTTC-3’; and GAPDH-F: 5’- GAACATCATCCCTGCCTCTAC-3’, GAPDH-R: 5’- CCTGCTTCACCACCTTCTT-3’. The PCR products were 330 bp for actin, 157 bp for HSP27, 152 bp for MYOIC and 182 bp for GAPDH. The PCR was undertaken using the following parameters: 95°C for 2 min, and 25 cycles of 95°C for 30s, 30s of 65°C for actin and GAPDH or 60°C for HSP27 and MYOIC and 72°C for 40s and final extension at 72°C for 7 min with DreamTaq DNA polymerase (Thermo Scientific, MA). Subsequently, the PCR products were analyzed by electrophoresis on 1.5% agarose gels.

### Statistical analysis

Data were analyzed using GraphPad Prism 5 program (GraphPad Software, Inc., CA). Statistical analysis of significance was undertaken by the paired sample test using SPSS (SPSS Inc.) with a *p*-value ≤0.05 taken as significant.

## Results

### Identification of DENV E protein interacting proteins

**Fig 2:**
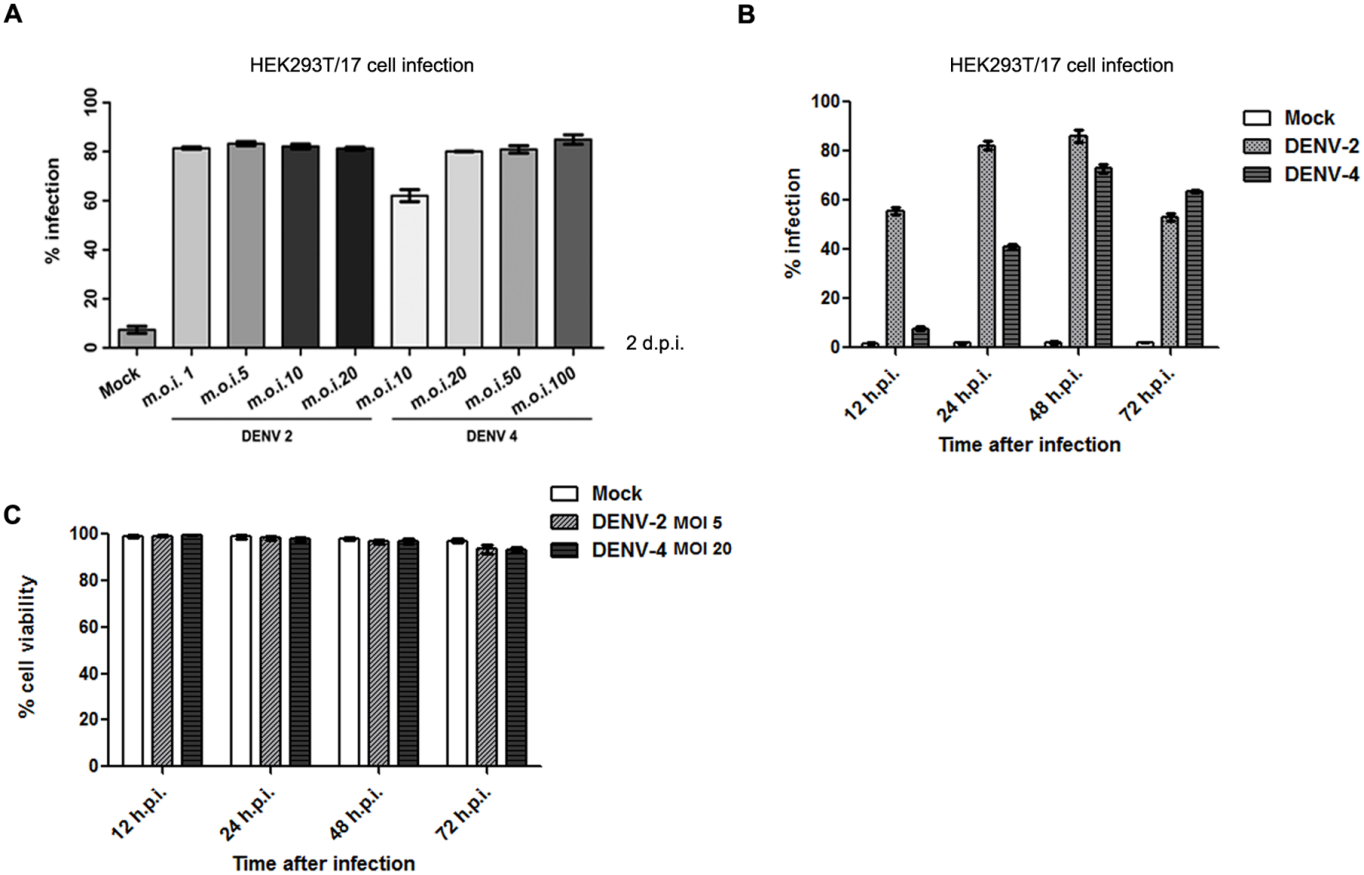
HEK293T cells were infected or mock infected with DENV 2 or DENV 4 under (A) different or (B) optimized m.o.i. and assessed for percentage infection by flow cytometry on (A) day 2 p.i. and (B) selected time points p.i. (C) Cell viability was determined for both DENV 2 and DENV 4 by trypan blue staining. Experiments were undertaken independently in triplicate. Error bars show ± S.E.M.

Our previous studies have shown that different DENVs show different infection levels after infection with the same m.o.i. [24, 25]. Therefore to optimize the infection of HEK293T/17 cells by DENV 2 and DENV 4, cells were infected with DENV 2 or DENV 4 at various multiplicity of infection (m.o.i.). HEK293T/17 was chosen as a model cell line as our previous work has shown this cell lines is both infectable with DENV [25] and transfectable with plasmid constructs [26], although as a cell line derived from human embryonic kidney [27] it is of uncertain clinical significance in the pathophysiology of dengue infection. The infected cells were collected at 2 d.p.i. and the degree of infection determined flow cytometry. Results showed that approximately equal levels of infection were seen with infection undertaken with m.o.i.s of 5 and 20 for DENV 2 and 4 respectively (Fig 2A), and these conditions were selected as standard conditions. A more detailed time course of infection was undertaken (Fig 2B) which showed an equal level of infection at 48 h.p.i., with DENV 2 showing higher infection levels at earlier time points (Fig 2). Cell viability was additionally assesses (Fig 2C) which showed no significant cell deficit as a consequence of infection.

**Fig 3:**
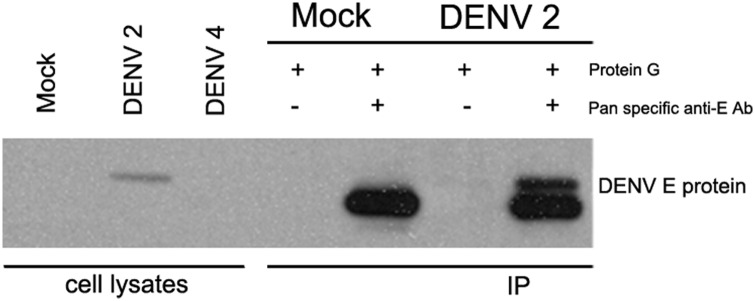
Western blot analysis of immunoprecipitated DENV E protein. HEK 293T/17 cells were mock infected or infected with DENV 2 and cell lysates were collected on day 2 p.i. Immunoprecipitation was performed using a pan specific anti-dengue E protein antibody and western analysis was undertaken with a different pan specific anti dengue E protein antibody. Co-immunoprecipitating proteins were identified on a parallel gels stained with silver (Fig 4).

To investigate cellular DENV E protein interacting proteins, HEK293T/17 cells were mock-infected or infected with DENV 2 and on day 2 post-infection DENV E protein was pulled down using a pan specific anti- dengue E protein antibody. Immunoprecipitates were separated by electrophoresis though SDS PAGE gels, and either directly subjected to silver staining or proteins were transferred to solid matrix support for western analysis with an anti-DENV E protein antibody to confirm immunoprecipitation. Results (Fig 3) showed that DENV 2 E protein was successfully immunoprecipitated. Parallel experiments with DENV 4 were undertaken, but the antibody used was unable to detect DENV 4 E protein in western analysis and so these experiments are not presented.

**Fig 4:**
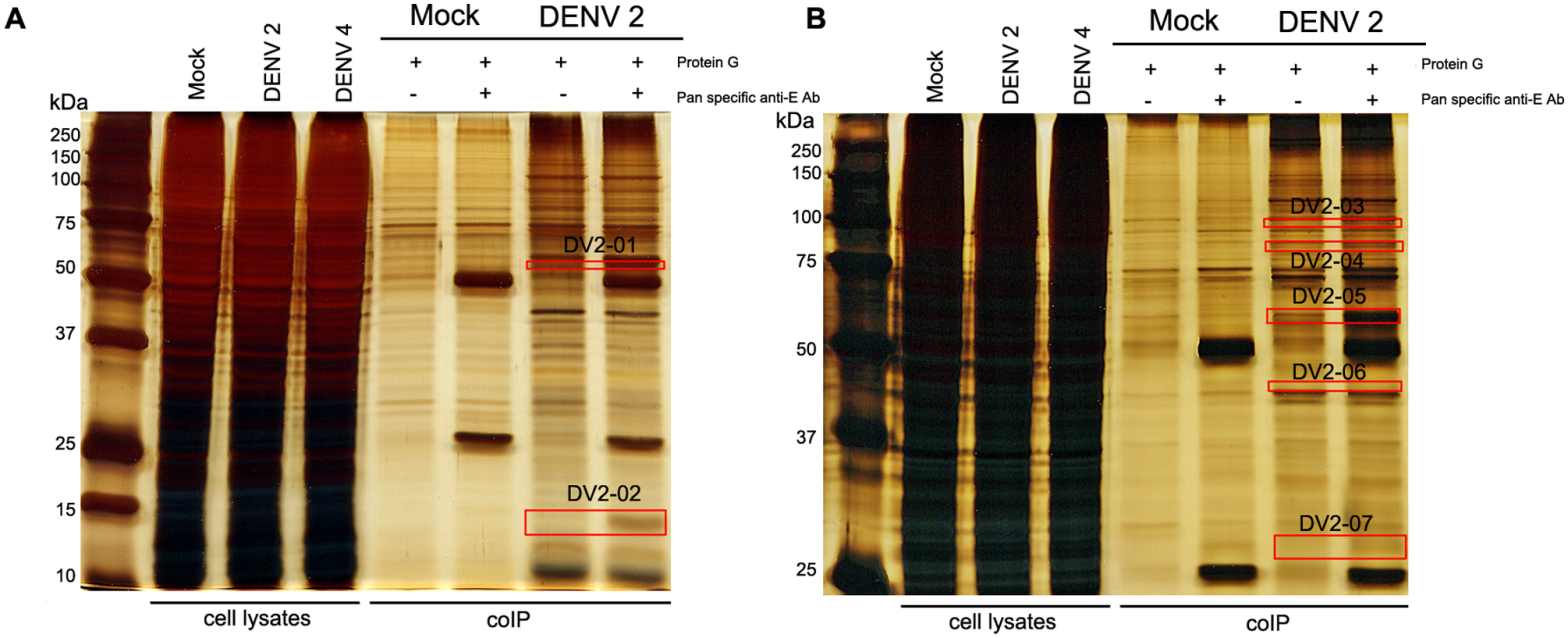
SDS-polyacrylamide gel electrophoresis of immunoprecipitated proteins. HEK 293T/17 cells were mock infected or infected with DENV 2 and cell lysates were collected at 24 hours post- transfection. Immunoprecipitation was performed using a pan specific anti-dengue E protein antibody and the immunoprecipitates were separated on (A) 12% and (B) 15% SDS–PAGE gels before silver staining. Boxed areas indicate bands that were excised for subsequent mass spectroscopy analysis. DENV 4 lysates are shown, but these experiments were discontinued as the antibody used in western analysis did not detect denatured DENV 4 E protein.

Protein bands that appeared to be specifically co-immunoprecipitated on the silver stained gels (Fig 4) were excised from the gels and subjected to protein identification through tryptic digestion and LC-MS/MS. Results identified a number of potential DENV E protein interacting partners, which included actin and a number of actin related proteins (Table 1).

**Table 1:** MS identified proteins from coIP reaction of DENV 2 infected cell lysate.

**Band DENV 2–01: size about 50 kDa**				
**Accession no.**	**Mass (Da)**	**Score**	**% coverage**	**Description**
gi 302634240	18890	78	6	Polyprotein (Dengue virus 2)
gi 259157602	55012	36	2	Envelope glycoprotein (Dengue virus 2)
**Band DENV 2–02: size about 15 kDa**				
gi 119603920	26306	16	4	Capping protein (actin filament) or Heat shock protein 27 (Hsp27)
**Band DENV 2–03: size about 120 kDa**				
gi 22477636	142000	26	2	TTBK1 protein (tau-tubulin kinase 1)
gi 12697929	110000	22	2	Ventricular zone expressed PH domain homolog 1
**Band DENV 2–04: size about 100 kDa**				
gi 119630257	110000	21	3	Myosin I
gi 20521986	110000	20	0	Sorting nexin 13
**Band DENV 2–05: size about 55 kDa**				
gi 19880695	42258	18	2	Actin-related protein T1
**Band DENV 2–06: size about 45 kDa**				
gi119619622	42404	28	2	Reticulocalbin, EF-hand calcium binding domain (in ER lumen)
gi 168480144	42258	24	2	β-actin
**Band DENV 2–07: size about 25 kDa**				
gi 12232485	20887	23	5	Interleukin-25 isoform 1 precursor
gi 229348	22222	20	4	Placental lactogen

### Interaction of DENV E protein and actin

**Fig 5:**
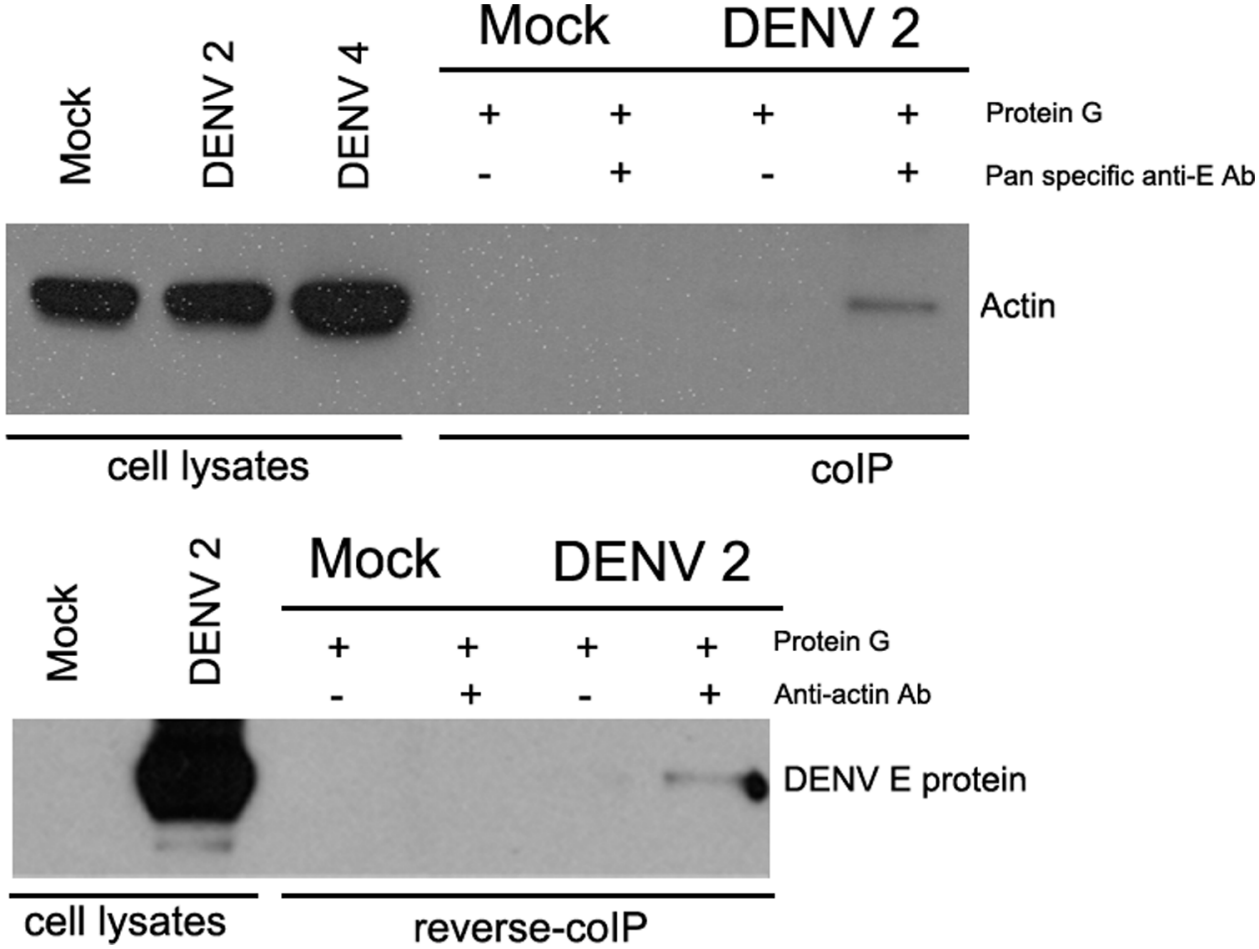
Western blot analysis of DENV 2 co-immunoprecipitated proteins. HEK 293T/17 cells were mock infected or infected with DENV 2 and cell lysates were collected on day 2 p.i. and (top) immunoprecipitation was performed using a pan specific anti-dengue E protein antibody and western analysis was undertaken with an anti-actin antibody or (bottom) immunoprecipitation was performed using an anti-actin antibody and western analysis was undertaken using a pan specific anti-dengue E protein antibody.

Although actin has previously been identified as a DENV E protein interacting protein [28], we sought to confirm the interaction. HEK293T/17 cells were therefore mock- infected or infected with DENV 2 and on day 2 p.i. DENV E protein was pulled down from the lysates using a pan specific anti-dengue E protein antibody, the immunoprecipitated proteins were electrophoretically separated and transferred to nitrocellulose membrane followed by western blot analysis using an anti-actin antibody. Results (Fig 5) indicated that actin was specifically co-immunoprecipitated with DENV 2 E protein. Reverse co-immunoprecipitation experiments using an anti-actin antibody confirmed the interaction between DENV 2 E protein and actin, (Fig 5).

As the antibody used was unable to detect DENV 4 E protein, and to determine whether DENV 4 E protein interacted with actin, eukaryotic expression vectors encoding flag peptide tagged-truncated E proteins (without the stem and transmembrane anchor regions) of DENV 2 and 4 (pcDNA-FLAG_D2ET, pcDNA-FLAG_D4ET) were constructed and transfected into HEK293T cells together with pcDNA-EGFP as a transfection control. Transfection efficiency was monitored by observation of transfected cells under an inverted fluorescence microscope on day 2 post-transfection using a primary anti-FLAG (anti-OctA-probe) primary antibody to detect the E protein constructs.

**Fig 6:**
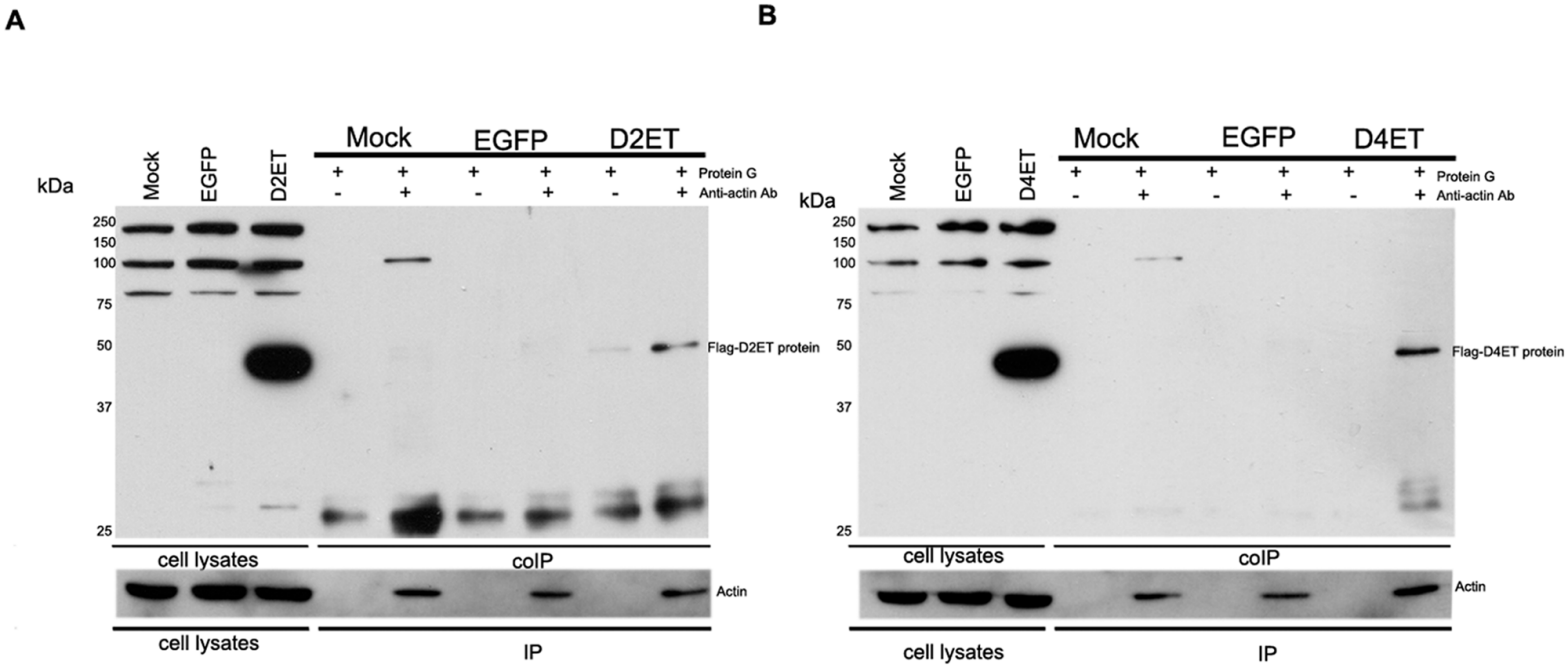
Western blot analysis of co-immunoprecipitated proteins (D2ET and D4ET). HEK 293T/17 cells were transfected with (top) D2ET or (bottom) D4ET and cell lysates were collected on day 2 post transfection. Immunoprecipitation was performed using an anti-actin antibody and western analysis was undertaken with an anti-FLAG (anti-OctA-Probe) antibody.

Lysates were prepared from transfected cells and actin immunoprecipitated using an anti-actin antibody. After electrophoretic separation and transfer to solid matrix support the immunoprecipitated proteins were subjected to western analysis with an anti-FLAG (anti-OctA-Probe) antibody. Results (Fig 6) showed the expected protein band size of about 43kDa for both FLAG-D2ET and FLAG-D4ET protein (Fig 6), demonstrating that actin was able to interact with both DENV 2 and DENV 4 E proteins.

### DENV E protein domains I and II interacts with actin

**Fig 7:**
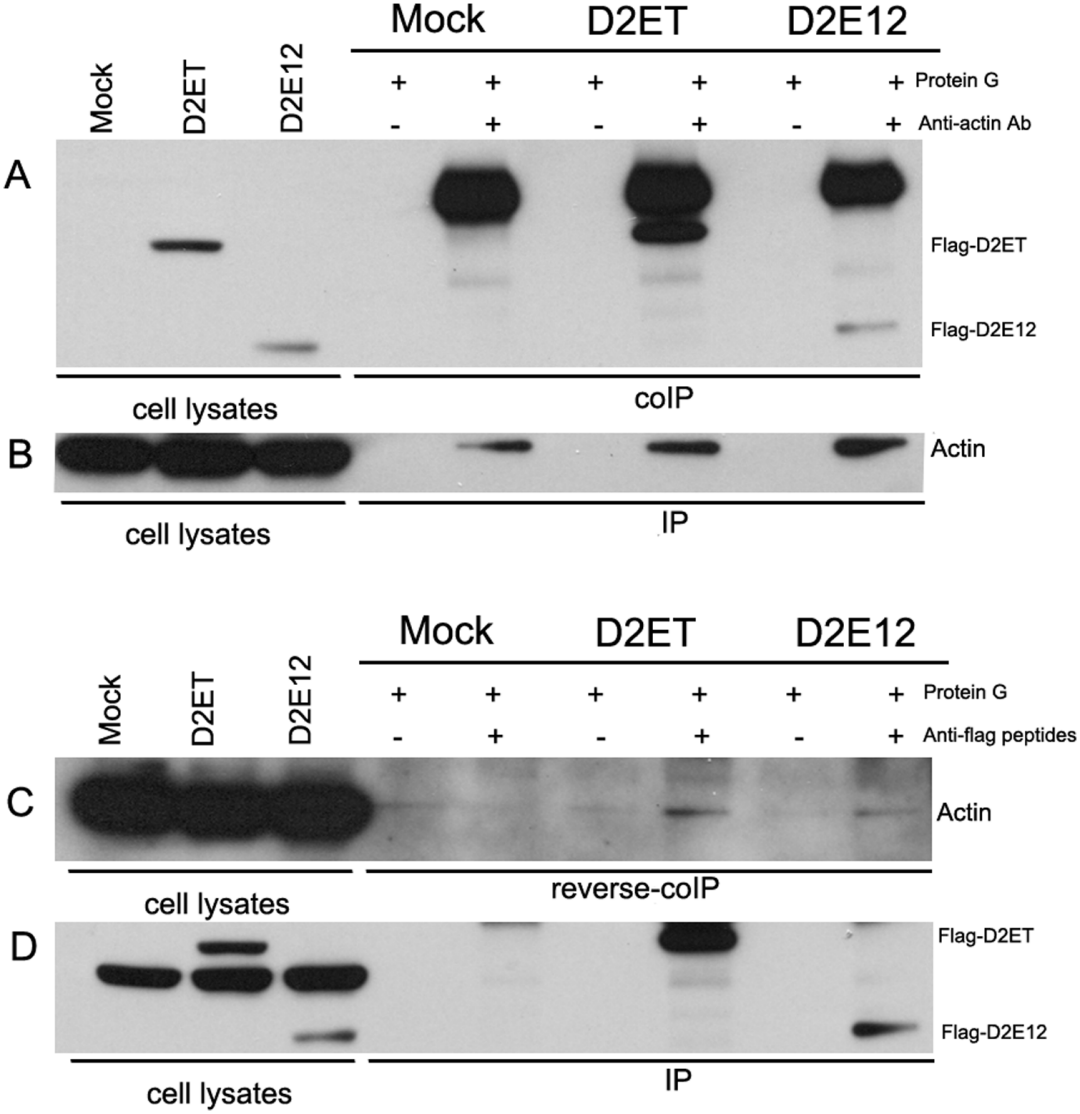
Western blot analysis of co-immunoprecipitated proteins (D2ET and D2E12). HEK 293T/17 cells were transfected with D2ET or D2E12 and cell lysates were collected on day 2 post transfection. (A) Immunoprecipitation was performed using an anti-actin antibody and western analysis was undertaken with an anti-FLAG (anti-OctA-Probe) antibody. (B) Filter from (A) was stripped and re-probed with an anti-actin antibody to confirm immunoprecipitation of actin. (C) Immunoprecipitation was performed using an anti-FLAG (anti-OctA-Probe) antibody and western analysis was undertaken with an anti-actin antibody. (D) Filter from (C) was stripped and re-probed with an anti-FLAG (anti-OctA-Probe) antibody to confirm immunoprecipitation of FLAG-tagged constructs.

To determine whether DENV E protein domain III (the primary antigenic domain and the domain responsible for receptor binding) mediated the interaction with actin, a DENV 2 protein construct containing only DENV E protein domains I and II was constructed (pcDNA-FLAG-D2E12). This construct was transfected into HEK293T/17 cells in parallel with FLAG-D2ET and co-immunoprecipitation and reverse co-immunoprecipitation experiments undertaken as previously described. The results (Fig 7) showed that an interaction between DENV 2 E protein and actin could be mediated by sequences present in domain I or II, in addition to those already identified in domain III [28].

### Co-localization between DENV E protein and actin

To investigate the expression of actin and DENV E protein and establish possible co-localization between these two proteins, HEK293T/17 cells were mock-infected or infected with DENV 2 or DENV 4 and cells were analyzed at several time points by confocal microscope after staining with an anti-dengue E protein antibody and phalloidin conjugated with TRITC for actin visualization in addition to a DAPI counterstain for nuclear visualization. Results (Figs A to D in S1 File) showed that the expression of E protein for both DENV 2 and 4 was maximal between 24 and 48 h.p.i, and that the level of expression decreased after this time. Analysis of colocalization of DENV E protein and actin showed partially co-localization in both DENV 2 and DENV 4 infected cells, with the highest level of co-localization being observed at 48 and 24 h.p.i. in DENV 2 and DENV 4 infected cells, respectively (Pearson correlation coefficient ± SD; CIs DENV 2 = 0.38± 0.01; ±0.01 and DENV 4 = 0.25 ± 0.03; ±0.03). There was a significant decrease in co-localization between actin and DENV E protein at 72 h.p.i. in both DENV 2 and DENV 4 infected cells (Pearson correlation coefficient ± SD; CIs 0.17 ± 0.018; ±0.02 and 0.07 ± 0.02; ±0.02, respectively) as compare to an average co-localization level at 12 to 48 h.p.i. (Pearson correlation coefficient ± SD DENV 2 = 0.35±0.035, DENV 4 = 0.23±0.032, *P* < 0.05). Furthermore, a relatively higher level of co-localization between DENV E protein and actin was observed in DENV 2 infected cells as compared to DENV 4-infected cells for each time point examined (Figs A to D in S1 File).

**Fig 8:**
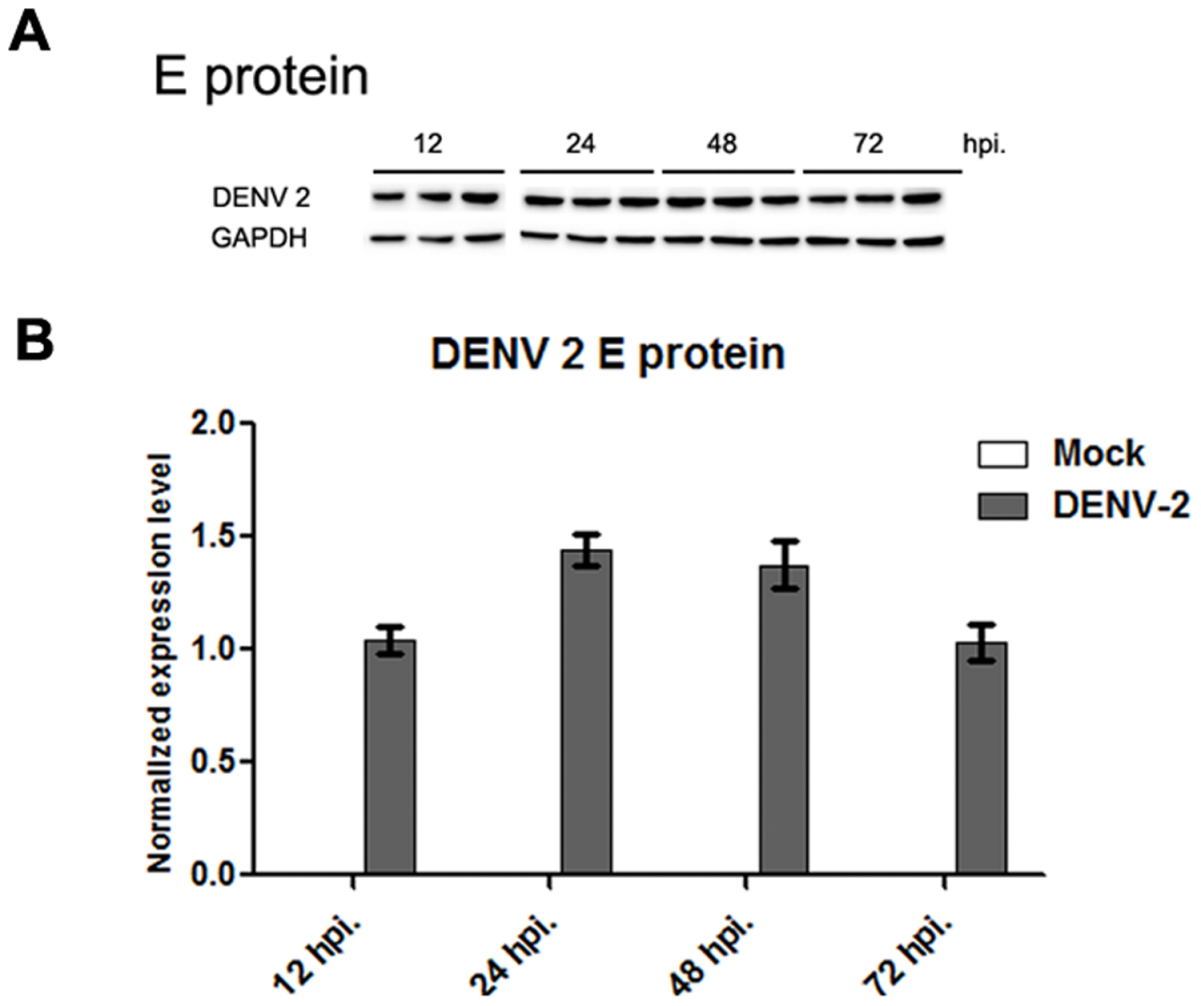
(A) HEK293T cells were mock-infected or infected with DENV 2 and protein lysates prepared at various time points after infection. Proteins subjected in to SDS-PAGE and western blot analysis undertaken using a pan specific anti-DENV E protein antibody and subsequently an anti-GAPDH antibody. Experiment was undertaken independently in triplicate. (B) Protein band intensities from (A) were measured using the ImageJ image analysis program and analyzed using the GraphPad Prism 5 program. Level of DENV E protein expression was normalized to GAPDH. Error bars show S.E.M.

The decrease of DENV E protein expression at 72 hours was confirmed for DENV 2 by Western blotting (Fig 8). As previous results had shown that DENV 4 E protein was not able to be detected in Western blots with the pan-specific antibody used, DENV 4 E protein expression was not determined by Western blotting.

**Fig 9:**
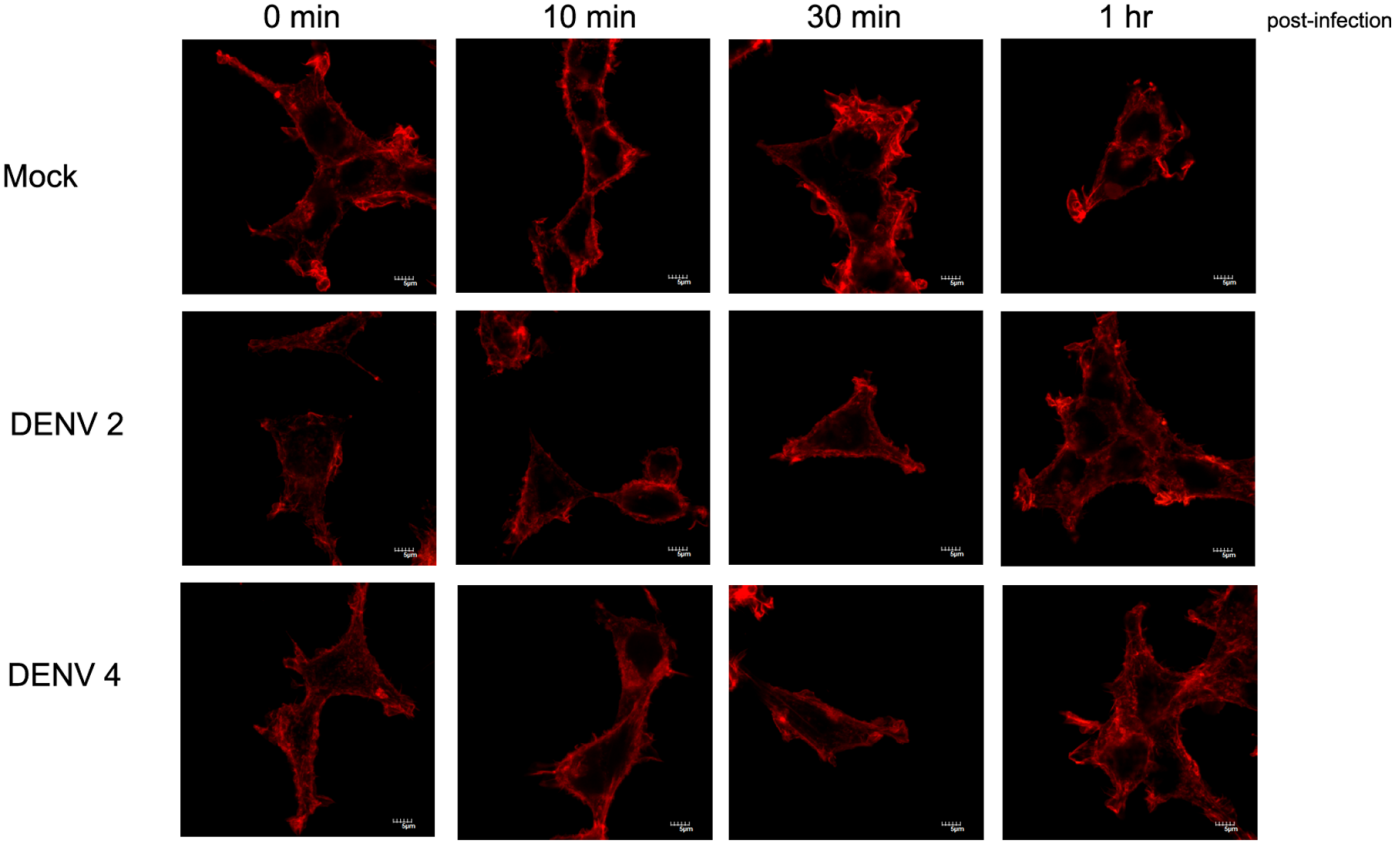
Analysis of actin expression. (A) HEK293T cells were mock-infected or infected with DENV 2 or DENV 4 and at 0, 20, 30 min and 1 hr p.i., cells were incubated with pan specific anti-dengue E protein antibody followed by an appropriate secondary antibody (green), phalloidin-TRITC (red) and DAPI (blue). Cells were observed under an Olympus Fluo View 1000 confocal microscope. Only the actin channel is shown. Full merged images with signal from all three channels are shown in Fig E in S1 File.

We additionally investigated actin expression during early infection to look for cytoskeletal re-organization. HEK293T/17 cells were therefore mock-infected or infected with DENV 2 or DENV 4 and cells were analyzed at 0, 20, 30 min, 1 hr as previously described. Results (Fig 9) showed no noticeable cytoskeletal rearrangement at time points soon after infection.

### Actin interacting proteins and DENV E protein

The original co-immunoprecipitation analysis identified some actin related proteins, namely myosin Ic and Hsp27. We were unable to verify any direct interaction between DENV E protein (with both DENV 2 and DENV 4 E proteins) and these proteins through co-immunoprecipitation analyses, although in one cases (Hsp27) the presence of non-specific bands rendered the results un-interpretable (Fig F in S1 File). However, it is likely that the presence of these proteins arises from their interaction with actin, rather than through an interaction with DENV E protein.

### Regulation of actin, myosin Ic and Hsp27 during DENV infection

**Fig 10:**
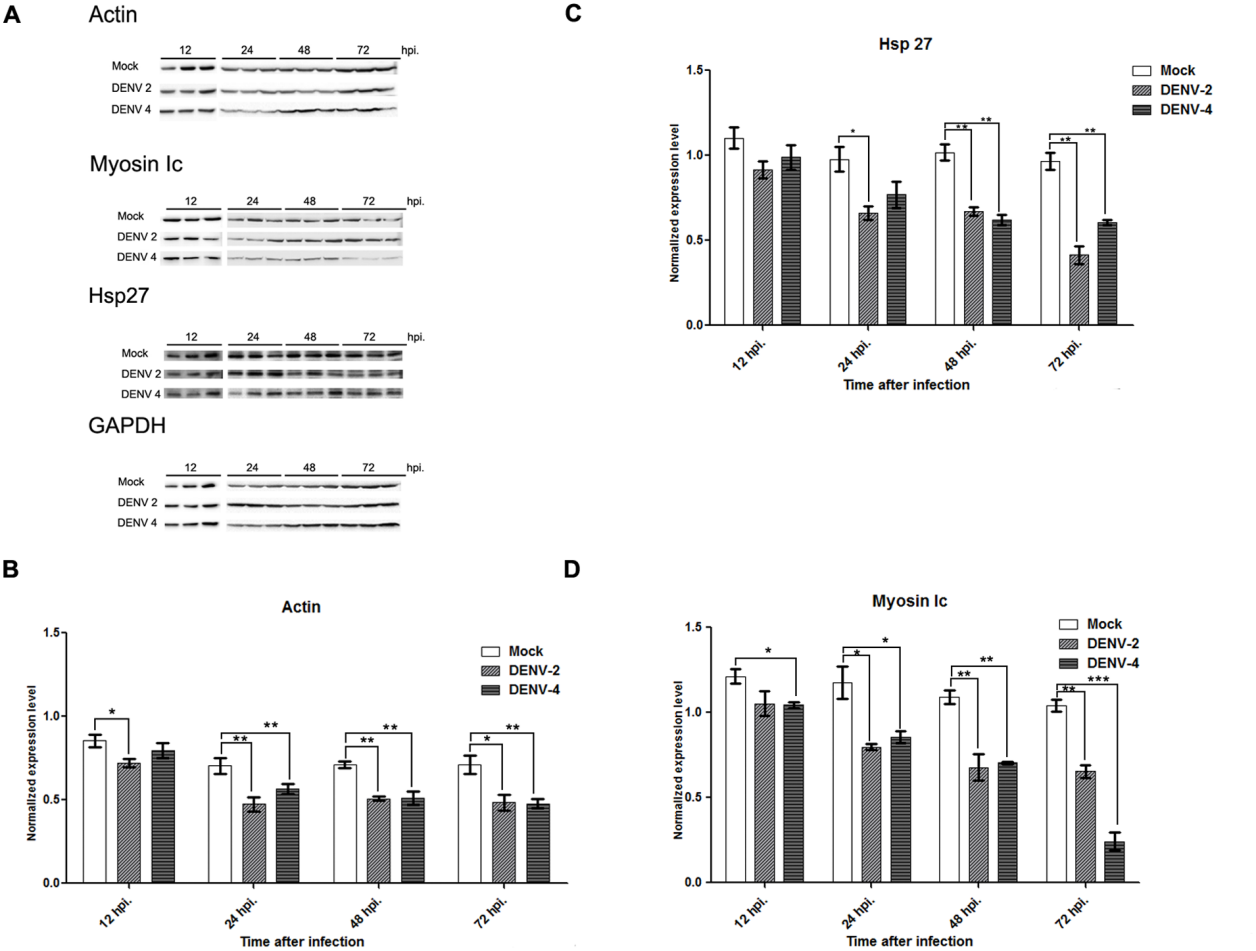
(A) HEK293T cells were mock-infected or infected with DENV 2 or DENV 4 and at the indicated time points protein lysates were collected, subjected to SDS-PAGE and western blot analysis using antibodies directed against actin, myosin Ic, Hsp27 and GAPDH. Protein band intensities were measured and normalized to GAPDH. Bar graphs show (B) normalized actin, (C) normalized myosin Ic and (D) normalized Hsp27. Experiments were undertaken independently in triplicate. Bars show ±S.E.M. *p<0.05, **p<0.01, ***p<0.001.

**Fig 11:**
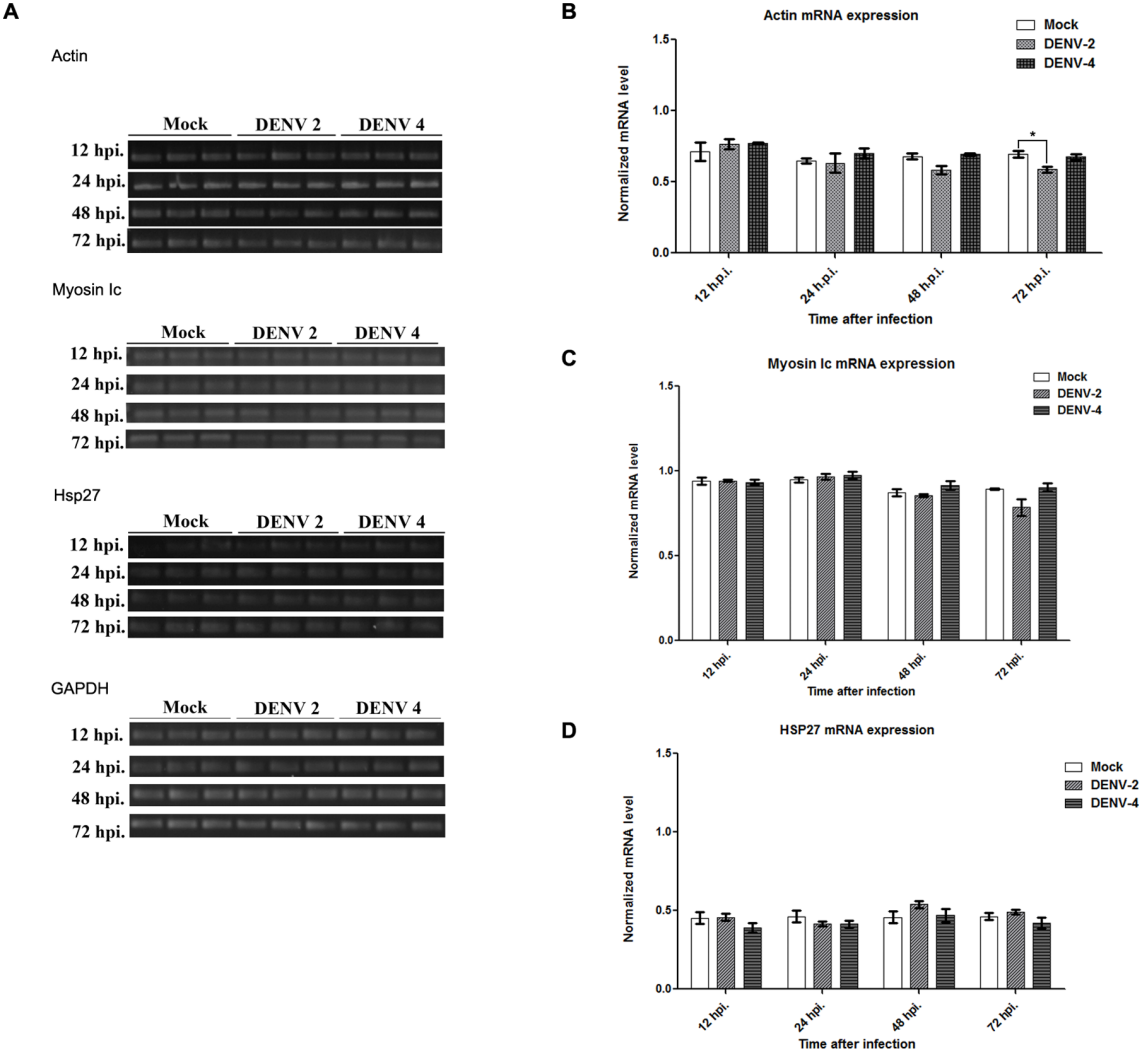
HEK293T cells were mock-infected or infected with DENV 2 or DENV 4 and at the indicated time points total RNA was extracted and used in (A) semi-quantitative RT-PCR to detect expression of actin, myosin Ic, Hsp27 and GAPDH. Experiments were undertaken independently in triplicate. Band intensities were measured using the ImageJ image analysis program and analyzed by the GraphPad Prism 5 program. The levels of mRNA expression was normalized against GAPDH. (B) normalized actin mRNA expression, (C) normalized myosin Ic mRNA expression and (D) normalized Hsp27 mRNA expression. Statistical analysis of significance as undertaken by the paired sample test using SPSS (SPSS Inc.) with a *p*-value ≤0.05 taken as significant. No significant differences were observed.

Although no direct interaction between DENV E protein and the identified actin interacting proteins was observed, the expression of myosin Ic and Hsp27 together with the expression of actin was investigated during DENV infection. HEK293T/17 cells were therefore mock-infected or infected with DENV 2 or DENV 4, and at selected time points protein expression was determined by western blot analysis, while mRNA expression levels were determined by semi-quantitative PCR. Results (Fig 10) show a significant reduction in actin protein levels during infection for both DENV 2 and DENV 4 infection starting from as early as 24 h.p.i. The reduction in actin protein expression is mirrored by in reductions in protein expression for both myosin Ic and Hsp27. Interestingly however, mRNA expression levels of all genes was relatively unaffected, with only a small reduction in actin mRNA levels being seen at 72 h.p.i for DENV 2 (Fig 11). No reduction in mRNA expression was observed for actin in DENV 4 infection or with either myosin Ic or Hsp27 in either DENV 2 or DENV infection.

## Discussion

The relatively small DENV genome encodes for only ten proteins [4]. These proteins must function to subvert the host cell innate immune responses and alter normal cellular functions to favour viral replication in addition to undertaking the process of viral replication directly through formation of the replication complex [14]. Evidence suggests therefore that the DENV proteins are multifunctional in that they target specific host cell processes in addition to their functions as structural proteins that will form the new virus progeny or non-structural proteins directly involved in viral replication. For example the DENV non-structural protein NS5 is the viral polymerase and methyltransferase, but additionally functions to shut down the host innate immunity interferon response through binding to STAT2 and promoting its degradation [29].

The DENV E protein is the major structural protein of the DENV virion [4] and the major antigenic determinant [30, 31]. The DENV E protein mediates virus receptor binding (REF) and a number of DENV E cell surface expressed receptor binding proteins have been identified (reviewed in [32, 33]). More recently an interactomic analysis of blood plasma proteins identified 27 plasma proteins capable of interacting with domain III of DENV E protein [34]. However, to date, only a few intracellular interactions between DENV E protein and cytosolic proteins have been described. These interactions include those with the ER resident chaperones GRP78 [10, 11, 15], calnexin and calreticulin [15] and actin [28]. This project sought to identify additional cellular E interacting proteins through co-immunoprecipitation. While several co-immunoprecipitating proteins were initially identified, we were only able to validate an interaction between DENV E protein and actin. As several of the other identified proteins were actin interacting proteins, it is likely that these were co-immunoprecipitated through the interaction with actin, and not with DENV E protein.

Our study therefore confirms the interaction between DENV E protein and actin as reported by Yang and colleagues [28]. We further observed that the interaction between DENV E and actin is not serotype specific as shown by the interaction between DENV 4 E protein and actin. However, in their study Yang and colleagues proposed that the domain mediating the interaction between E protein and actin resides in the DENV E protein domain III [28]. As shown here however, a construct containing only domains I and II of DENV E protein was capable of interacting with actin, suggesting the existence of multiple actin interacting domains in the DENV E protein.

Yang and colleagues additionally observed highest colocalization between actin and DENV E protein at 96 h.p.i. in infected endothelial cells [28]. In the present study maximal colocalization was seen between 24 and 48 h.p.i, with a decline in colocalization after this time. Importantly, western analysis showed significantly decreased expression of actin by 72h.p.i. consistent with the reports of other authors of a decrease in actin expression in DENV infected endothelial cells [35]. Interestingly, we observed no actin reorganization upon DENV infection in HEK293T cells which contrasts to the actin reorganization observed in DENV infected endothelial cells [28, 35], and suggests that there may be cell type specific differences in the process of cytoskeletal re-arrangement during DENV infection.

The decline in actin expression observed in western analysis was largely in the absence of a reduction in transcription, although a slight decline in mRNA levels were seen in DENV 2 infection at 72 h.p.i. Interestingly the other actin associated genes examined also showed significant reductions in protein levels during the course of infection, but no reduction in transcription. This suggests that the decline in protein levels seen with actin and other actin associated proteins occurs through a primarily post-translational or degradative process, rather than by transcriptional regulation.

It has been suggested that the association between actin and DENV E protein occurs at several stages of the replication cycle [28]. It is known that actin is required during the viral entry process [36], and disruption of actin results in a reduction of virus entry and release [36], however it remains to be shown whether there is a direct interaction at these stages of the replication cycle. Similarly others have suggested that the primary interaction occurs at a late time point in infection and that the interaction mediates viral egress [28]. Again, a specific interaction between actin and DENV E protein at viral release remains to be shown. Our results show maximum colocalization at 24–48 h.p.i. and decreased protein expression starting from 24 h.p.i would seem to be inconsistent with the actin: DENV E protein interaction having a major role in viral egress.

The intent of this study was to identify additional cellular proteins that interact with DENV E protein, and the results were somewhat disappointing in that the candidate we identified and validated was actin, a protein previously identified by others [28]. Interestingly a large yeast-two hybrid screen failed to identify any DENV E interacting proteins, despite the identification of numerous interacting proteins for other DENV proteins [37]. While the authors of that study proposed the failure to identify any DENV E interacting proteins was a result of the experimental system used [37], collectively it is possible that DENV E protein has relatively few cellular interacting partners.

## Supporting Information

S1 File Contains the following files: **Fig A.** Co-localization analysis of DENV E protein and actin (12 h.p.i). **Fig B.** Co-localization analysis of DENV E protein and actin (24 h.p.i). **Fig C.** Co-localization analysis of DENV E protein and actin (48 h.p.i). **Fig D.** Co-localization analysis of DENV E protein and actin (72 h.p.i). **Fig E.** Co-localization analysis of DENV E protein and actin (early time points). **Fig F.** Co-immunoprecipitation analysis proteins myosin 1c and hsp27).
(PDF)
