# Supplementary material for: Actin Interacts with Dengue Virus 2 and 4 Envelope Proteins
Source: PLoS One. 2016 Mar 24;11(3):e0151951. doi: 10.1371/journal.pone.0151951 (PMC4806980; doi:10.1371/journal.pone.0151951)
Supplement: S1 File — Contains the following files: Fig A. Co-localization analysis of DENV E protein and actin (12 h.p.i). Fig B. Co-localization analysis of DENV E protein and actin (24 h.p.i). Fig C. Co-localization analysis of DENV E protein and actin (48 h.p.i). Fig D. Co-localization analysis of DENV E protein and actin (72 h.p.i). Fig E. Co-localization analysis of DENV E protein and actin (early time points). Fig F. Co-immunoprecipitation analysis proteins myosin 1c and hsp27). (PDF) [file pone.0151951.s001.pdf]

S1 File: Supplemental materials

Actin interacts with dengue virus 2 and 4 envelope proteins

Kunlakanya Jitoboam[1], Narumon Phaonakrop[2], Sirikwan Libsittikuk[1], Chutima Thepparit[1], Sittiruk Roytrakul[2], Duncan R. Smith[1,3]

[1]Institute of Molecular Biosciences, Mahidol University, Salaya campus, 25/25 Phuttamonton Sai 4, Salaya, Nakorn Pathom, Thailand 73170

[2]National Center for Genetic Engineering and Biotechnology (BIOTEC), National Science and Technology Development Agency, 113 Thailand Science Park, Phahonyothin Road, Khlong Nueng, Khlong Luang, Pathum Thani, Thailand 12120

[3]Center for Emerging and Neglected Infectious Diseases, Mahidol University, Salaya campus, 25/25 Phuttamonton Sai 4, Salaya, Nakorn Pathom, Thailand 73170

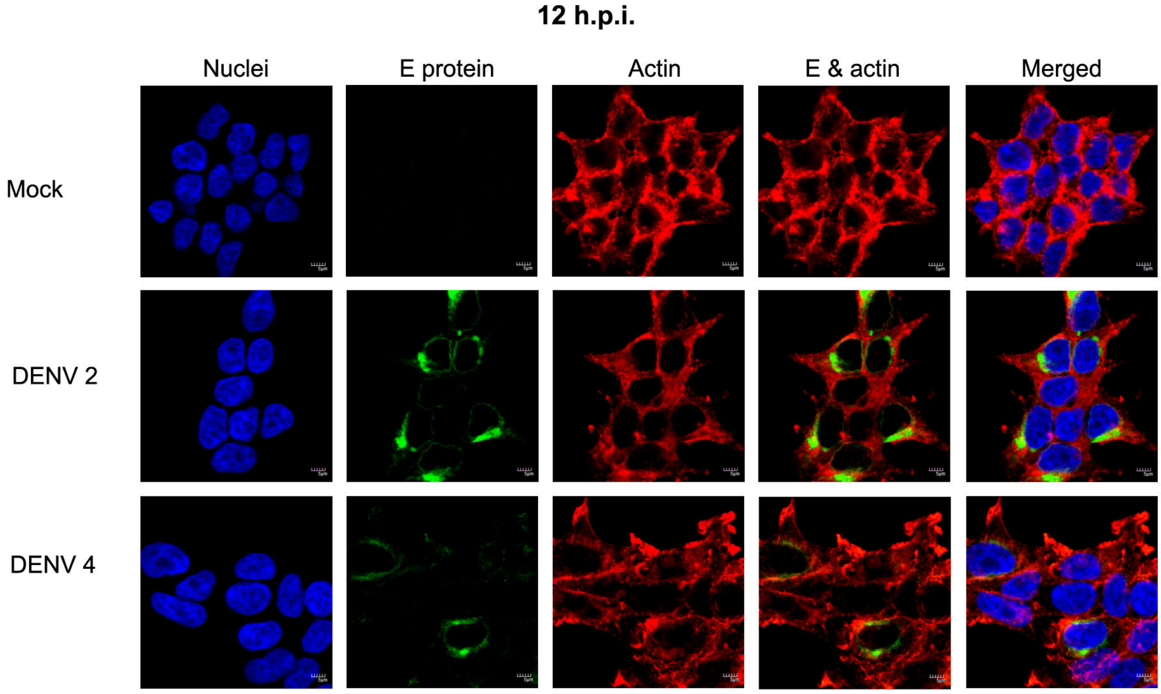

Figure A. Co-localization analysis of DENV E protein and actin (12 h.p.i).

HEK293T cells were mock-infected or infected with DENV 2 or DENV 4 and at 12, h.p.i. cells were incubated with pan specific anti-dengue E protein antibody followed by an appropriate secondary antibody (green), phalloidin-TRITC (red) and DAPI (blue). Cells were observed under an Olympus Fluo View 1000 confocal microscope. Representative individual and merged images are shown.

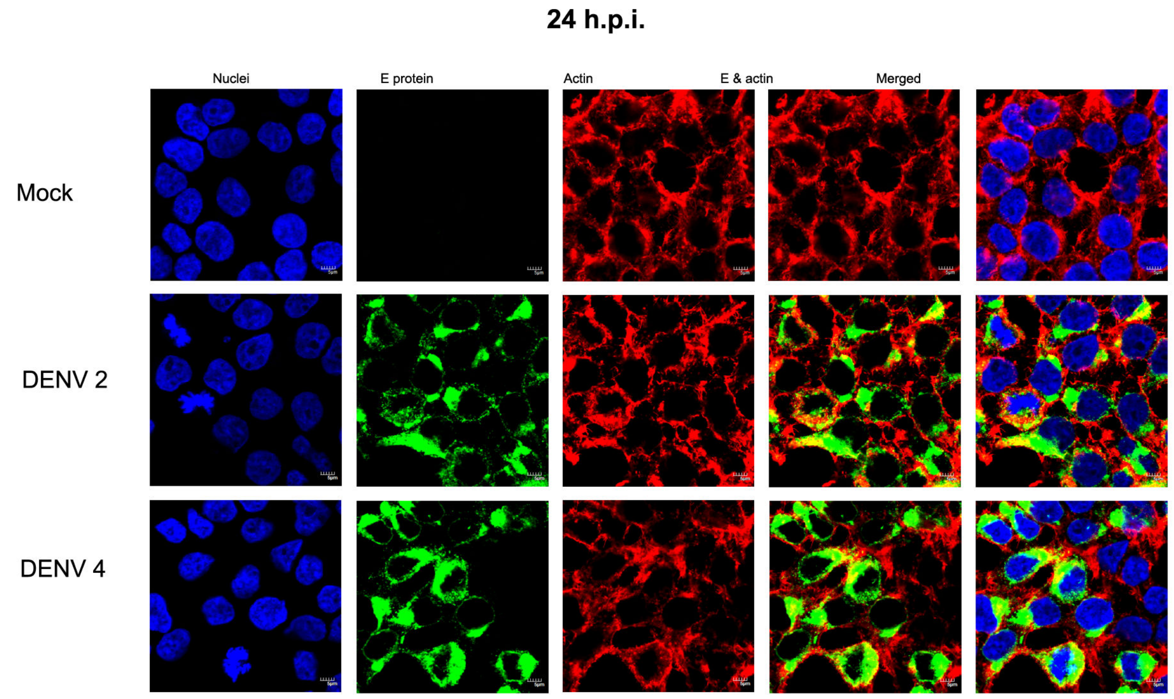

Figure B. Co-localization analysis of DENV E protein and actin (24 h.p.i).

HEK293T cells were mock-infected or infected with DENV 2 or DENV 4 and at 24 h.p.i., cells were incubated with pan specific anti-dengue E protein antibody followed by an appropriate secondary antibody (green), phalloidin-TRITC (red) and DAPI (blue). Cells were observed under an Olympus Fluo View 1000 confocal microscope. Representative individual and merged images are shown.

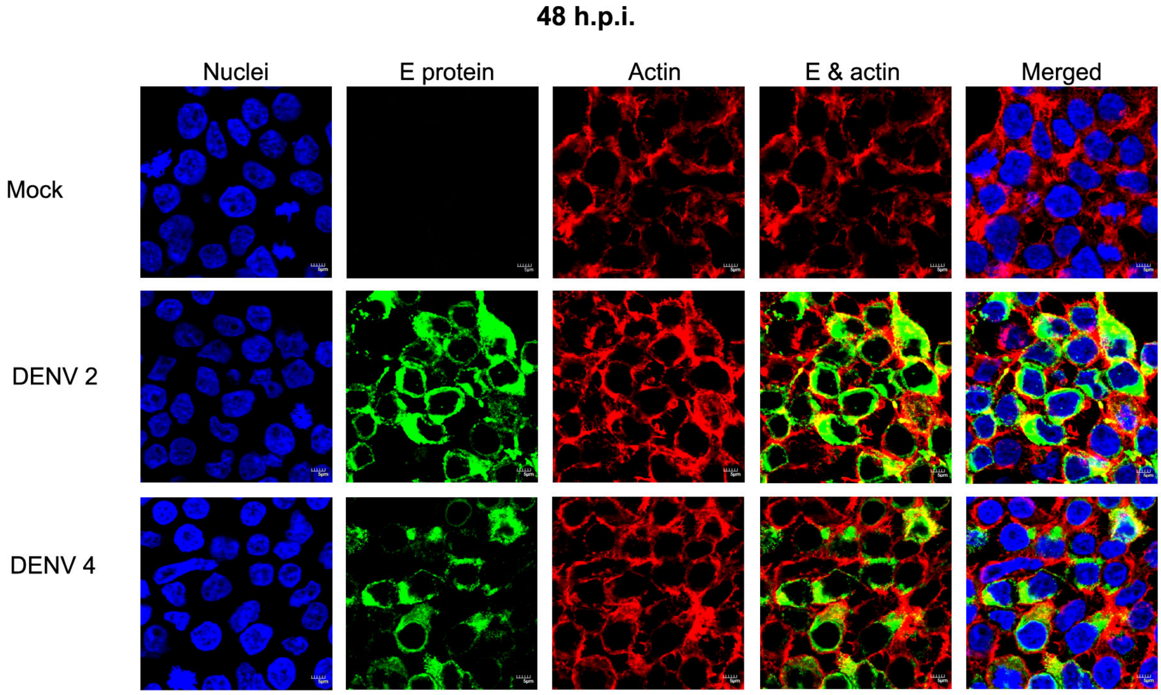

Figure C. Co-localization analysis of DENV E protein and actin (48 h.p.i).

HEK293T cells were mock-infected or infected with DENV 2 or DENV 4 and at 48 h.p.i. cells were incubated with pan specific anti-dengue E protein antibody followed by an appropriate secondary antibody (green), phalloidin-TRITC (red) and DAPI (blue). Cells were observed under an Olympus Fluo View 1000 confocal microscope. Representative individual and merged images are shown.

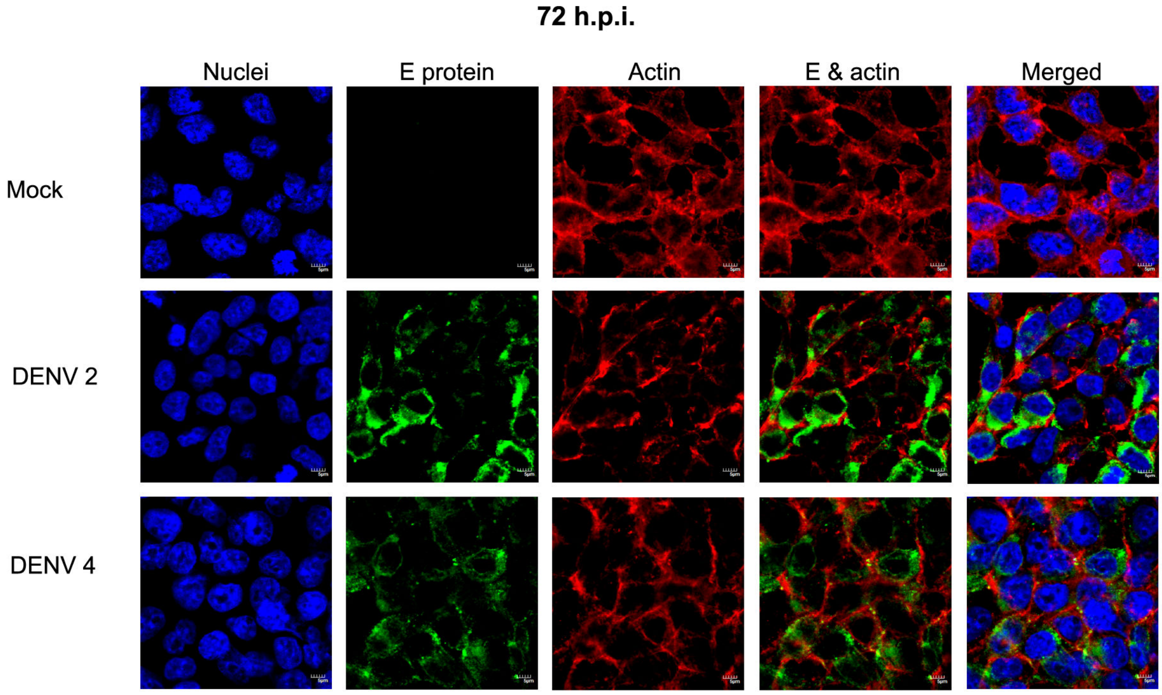

Figure D. Co-localization analysis of DENV E protein and actin (72 h.p.i).

HEK293T cells were mock-infected or infected with DENV 2 or DENV 4 and at and 72 h.p.i., cells were incubated with pan specific anti-dengue E protein antibody followed by an appropriate secondary antibody (green), phalloidin-TRITC (red) and DAPI (blue). Cells were observed under an Olympus Fluo View 1000 confocal microscope. Representative individual and merged images are shown.

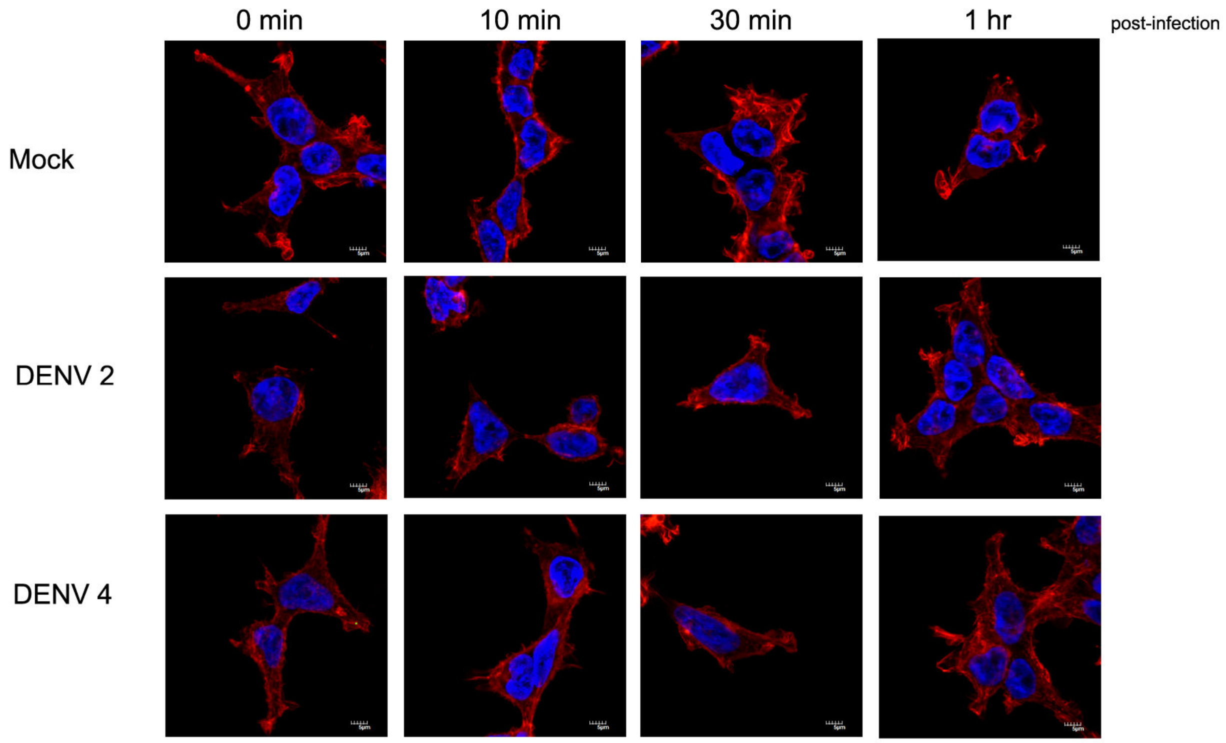

Figure E. Co-localization analysis of DENV E protein and actin (early time points) .

HEK293T cells were mock-infected or infected with DENV 2 or DENV 4 and at 0, 20, 30 min and 1 hr p.i., cells were incubated with pan specific anti-dengue E protein antibody followed by an appropriate secondary antibody (green), phalloidin-TRITC (red) and DAPI (blue). Cells were observed under an Olympus Fluo View 1000 confocal microscope. Representative merged images are shown. The isolated actin channel is shown in Figure 9 of the main article.

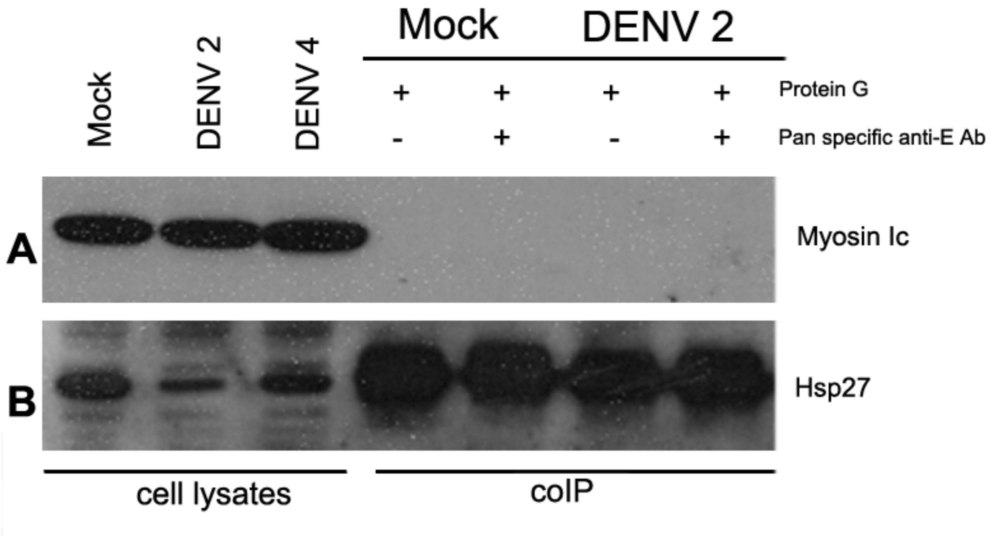

Figure F. Co-immunoprecipitation analysis proteins myosin 1c and hsp27).

HEK 293T/17 cells were mock infected or infected with DENV 2 and cell lysates were collected on day 2 p.i. immunoprecipitation was performed using a pan specific anti-dengue E protein antibody and western analysis was undertaken with an (A) an anti-myosin 1c antibody and (B) an anti-hsp27 antibody.
